# The Role of Artificial Intelligence in Triaging Patients in Eye Casualty Departments: A Systematic Review

**DOI:** 10.7759/cureus.78144

**Published:** 2025-01-28

**Authors:** Aaruran Nadarajasundaram, Simeon Harrow

**Affiliations:** 1 Emergency Department, St Thomas' Hospital, London, GBR; 2 Emergency Medicine Department, Maidstone and Tunbridge Wells NHS Trust, Maidstone, GBR

**Keywords:** artificial intelligence, eye casualty, eye disease, innovation, triaging

## Abstract

Visual impairment and eye disease remain a significant burden, highlighting the need for further support regarding eye care services. Artificial intelligence (AI) and its rapid advancements are providing an avenue for transforming healthcare. As a result, this provides a potential avenue to address the growing challenges with eye health and could assist in settings such as eye casualty departments. This review aims to evaluate current studies on AI implementation in eye casualty triage to understand the potential application for the future. A systematic review was conducted across a range of sources and databases producing 77 records initially identified, with four studies included in the final analysis. The findings demonstrated that AI tools are able to produce consistent and accurate triaging of patients and provide improvement in work efficiency without compromising safety. However, we note limitations of the studies including limited external validations of results and general applicability at present. Additionally, all the studies highlight the need for further studies and testing to allow for better understanding and validation of AI tools in eye casualty triaging.

## Introduction and background

Eye casualty departments play a crucial role in managing acute ocular conditions that require immediate attention. In the United Kingdom, these departments experience a significant volume of attendance, with estimates ranging from 20 to 30 per 1,000 population annually [1]. This substantial demand highlights the importance of efficient triage and management systems to ensure timely and effective patient care. Triage is defined as the process by which healthcare professionals assess the severity and order of priority for providing treatment for individuals. With the growing demand for eye-related diseases, optimizing the efficiency of this process would allow for a greater number of patients to be treated. Commonly, triaging relies on assessment and details of referrals with subsequent re-assessment by the eye casualty department. Artificial intelligence (AI) can utilize data and modelling to help improve efficient triaging and improve patient care efficiency.

Globally, visual impairments and eye disease contribute significantly to the burden of disease. According to the World Health Organization (WHO), at least 2.2 billion people have a near or distant vision impairment, with at least one billion cases being preventable or yet to be addressed [2]. The leading causes of vision impairment include uncorrected refractive errors and cataracts, both of which are prevalent across various regions and demographics. The Global Burden of Disease Study 2021 further highlights the impact of vision loss on global health. The study estimated that 1.1 billion people were living with vision impairment, including blindness, with a substantial proportion of these cases being preventable or treatable [3,4].

This rising trend emphasizes the need for enhanced eye care services and preventive measures to address the growing incidence of visual impairments and their associated health burdens. In recent years, AI and machine learning (ML) systems have been developed for applications in the medical field. ML, which forms a subdivision of AI, can utilize algorithms to make predictions from the patterns observed when analyzing patient data and be mapped against an output [5]. The current implementation of AI in ophthalmology is predominantly in disease detection through the analysis of imaging modalities, with success noted in diabetic retinopathy, retinopathy of prematurity, age-related macular degeneration and glaucoma [6-9]. However, to date, there remains a paucity in the literature involving the potential implementation of AI for triaging patients in the context of eye casualty.

This review seeks to analyze and evaluate the current studies regarding the implementation of AI in triaging within eye casualty settings, to compare service efficiency and patient safety. Through examination of the currently available evidence, this systematic review highlights the potential scope for the implementation of AI for triaging patients within eye casualty departments to improve efficiency and service quality.

## Review

Methodology

This review was reported using Preferred Reporting Items for Systematic Reviews and Meta-Analyses (PRISMA) 2020 guidelines [10].

Search Strategy

A comprehensive literature search was conducted through multiple electronic databases, including PubMed, Cochrane Library, Google Scholar, Web of Science, Embase and Scopus. The search strategy employed a Boolean search method with a combination of keywords and Medical Subject Headings (MeSH) terms. The terms used in PubMed, Google Scholar and Web of Science were "artificial intelligence" "machine learning" OR "machine learning" OR "deep learning" OR "AI" AND "ophthalmology", "eye diseases" OR "eye casualty" AND "Innovation" OR "implementation" OR "current roles" OR "AI tools".

Boolean search strings utilized in Cochrane Library were ("Artificial Intelligence" OR "Machine Learning" OR "artificial intelligence" OR "AI" OR "machine learning") AND ("Ophthalmology" OR "Eye Diseases" OR "eye casualty") AND ("Innovation" OR "implementation" OR "current roles" OR "AI tools").

Embase search was performed using ('artificial intelligence'/exp OR 'machine learning'/exp OR 'artificial intelligence' OR 'AI' OR 'machine learning') AND ('ophthalmology'/exp OR 'eye disease'/exp OR 'eye casualty' OR 'ophthalmology' OR 'eye emergency') AND ('innovation' OR 'current roles' OR 'AI tools').

Scopus search was performed using (TITLE-ABS-KEY("artificial intelligence" OR "AI" OR "machine learning")) AND (TITLE-ABS-KEY("ophthalmology" OR "eye diseases" OR "eye casualty" OR "eye emergency")) AND (TITLE-ABS-KEY("innovation" OR "current roles" OR "AI tools")).

The search was limited to English-language publications from 2020 to November 2024 to evaluate the increased implementation of AI in healthcare following the COVID-19 pandemic. Furthermore, reference lists of relevant articles and reviews were manually screened to identify any potentially missed studies which would be excluded from the review.

Additionally, clinicaltrial.gov was reviewed for any potential conducted trials relevant to the review using the Boolean search string: "artificial intelligence" OR "AI" OR "machine learning" AND "ophthalmology" OR "eye diseases" OR "eye casualty" OR "eye emergency" AND "innovation" OR "current roles" OR "AI tools".

Inclusion and Exclusion Criteria

Two independent reviewers (AN and SH) screened the titles and abstracts of the retrieved articles for eligibility. Inclusion criteria were based upon the following pre-defined criteria: validity or mixed methods studies, patients attending eye casualty departments in hospital settings, and comparison of efficiency and accuracy of AI triaging compared to current practice. This systematic review focused on mixed method and validation studies as these were the only study methods at present, where the use of AI in eye casualty patient triaging was evaluated.

The inclusion criteria included limiting our search to full-text English language-only studies. Exclusion criteria consisted of review articles, case reports, case series and editorials. Articles with missing or incomplete data were also omitted from our study.

Data Collection

**Figure 1:**
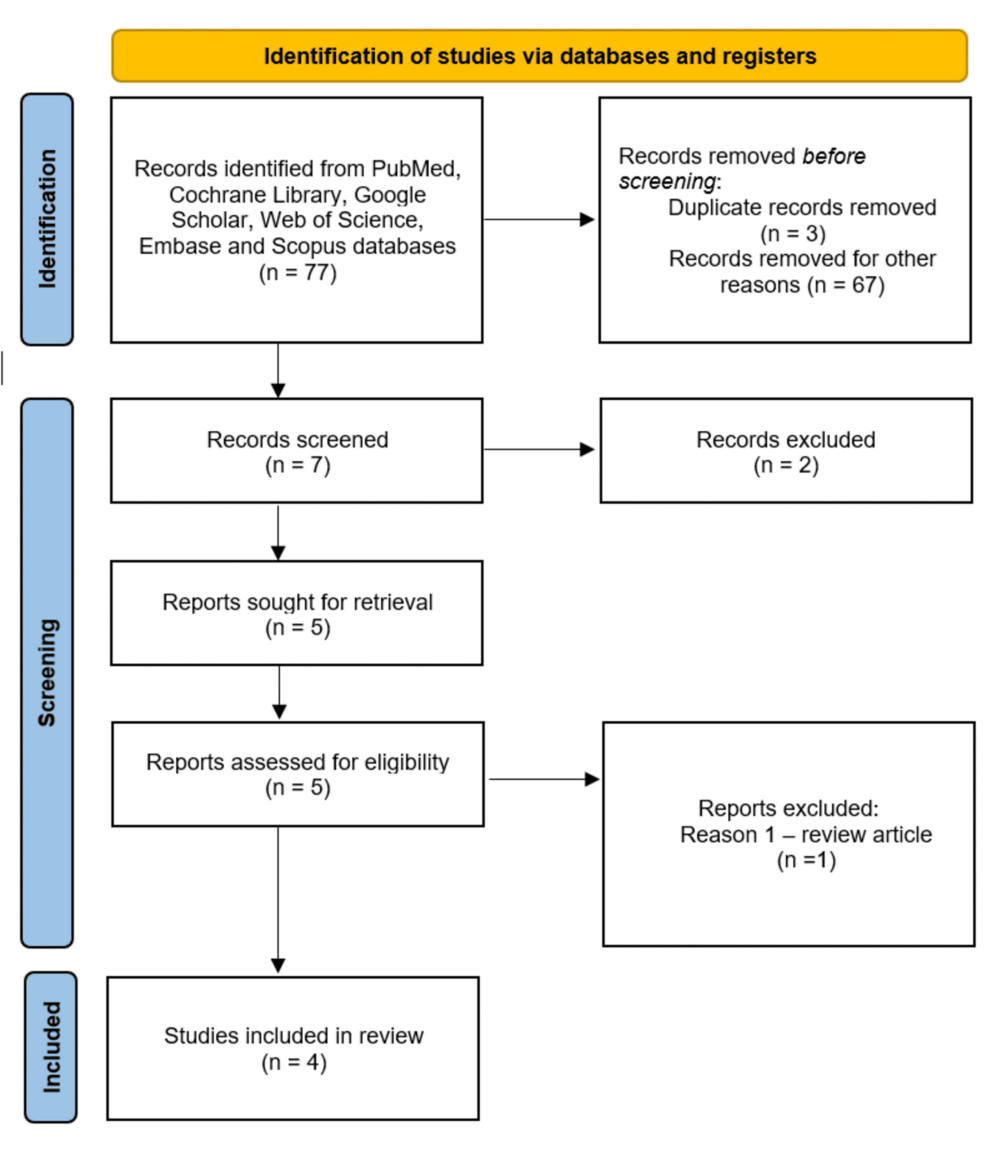
PRISMA flowchart representing the study selection process. PRISMA: Preferred Reporting Items for Systematic Reviews and Meta-Analyses

Our search produced four eligible studies from which data was extracted using the CHecklist for critical Appraisal and data extraction for systematic Reviews of prediction Modelling Studies (CHARMS) tool to ensure all relevant data was captured [11]. Extracted data was added and analyzed using Microsoft Excel software (Microsoft® Corp., Redmond, WA, USA). The process of selection of relevant studies was documented using a PRISMA flow diagram (Figure 1).

A comparative evaluation of the key findings of each of the four studies for our review question is highlighted in Table 1.

**Table 1:** Comparative evaluation of four studies included in the systematic review. AUC: area under the receiver operating characteristic curve

Study number	Study name	Author(s) and year	Dataset size	Type of study	Outcome measures used	AI system/tool used	Outcomes/results	Key findings on implementing AI in eye casualty settings
1	EE-Explorer: A Multimodal Artificial Intelligence System for Eye Emergency Triage and Primary Diagnosis	Chen et al. 2023 [12]	2,405 internal; 103 external	Model validation and reliability study	Triage classification (urgent, semiurgent, nonurgent)	DenseNet201 & XGBoost	AUC = 0.982 (internal); AUC = 0.988 (external)	Demonstrated high accuracy in triage classification; limited by small external validation dataset and reliance on advanced imaging modalities.
2	Usability of an Artificially Intelligence-Powered Triage Platform for Adult Ophthalmic Emergencies: A Mixed Methods Study	Jindal et al. 2023 [13]	20 participants	Mixed methods usability study	User acceptance, usability, and safety metrics	DOTS (AI triage platform)	High user acceptance (95%) of user willingness to incorporate platform in clinical workflow.	Highlighted potential for user-friendly AI platforms but emphasized need for larger-scale validation.
3	A Machine Learning System to Optimise Triage in an Adult Ophthalmic Emergency Department: A Model Development and Validation Study	Brandao-de-Resende et al. 2023 [14]	1269 (internal); 761 (external)	Model development and validation	Triage categories (emergency, urgent, elective)	DOTS, XGBoost	Sensitivity = 95.2% (external validation); specificities lower in non-urgent cases. DOTS exhibited consistent performance at both internal and external validation and was robust to varying relative disease incidences.	High sensitivity supports safe triage; further validation needed for optimizing specificity in non-urgent cases.
4	Artificial Intelligence Method to Classify Ophthalmic Emergency Severity Based on Symptoms: A Validation Study	Ahn 2020 [15]	1,681 (retrospective)	Model validation study	Severity categories (immediate, urgent, semi-urgent, non-urgent)	Ensemble neural network model	Accuracy = 99.05% in internal validation; SMOTE used to balance class distribution.	Demonstrated robust accuracy in severity classification; potential logistical challenges in real-world implementation.

Subsequently, the risk of bias was assessed utilizing the Prediction Model Risk of Bias Assessment Tool (PROBAST) to assess the risk of bias (ROB) and the applicability of each of the four studies reviewed (Table 2) [11].

**Table 2:** PROBAST ROB and ROA findings of the eligible studies for this systematic review. PROBAST: Prediction Model Risk of Bias Assessment Tool; ROB: risk of bias; ROA: risk of applicability

Study name	Participants	Predictors	Outcome	Analysis	Applicability concern	Justification
EE-Explorer: A Multimodal Artificial Intelligence System for Eye Emergency Triage and Primary Diagnosis [12]	Low	Low	Low	High	High	The study demonstrated high accuracy and sensitivity in internal and limited external validation. However, the small external dataset and reliance on advanced imaging tools raise high concerns about scalability and generalisability.
Usability of an Artificially Intelligence-Powered Triage Platform for Adult Ophthalmic Emergencies: A Mixed Methods Study [13]	Low	Low	Low	High	High	Focused on usability and clinician feedback, the study lacked performance metrics, external validation, and large-scale testing, making its direct applicability to AI implementation in eye casualties limited.
A Machine Learning System to Optimise Triage in an Adult Ophthalmic Emergency Department: A Model Development and Validation Study [14]	Low	Low	Low	Low	Low	The study included a large, diverse dataset, robust validation, and operational insights, demonstrating a strong potential for AI triage implementation in eye casualty settings.
Artificial Intelligence Method to Classify Ophthalmic Emergency Severity Based on Symptoms: A Validation Study [15]	High	Low	Low	High	High	Despite strong internal validation, the study lacked external validation and practical implementation strategies, raising high concerns about its scalability and use in broader settings.

Discussion

AI has emerged as a transformative tool in healthcare, particularly in ophthalmology, where its applications range from diagnostics to patient triage. In eye casualty settings, AI triage systems aim to enhance departmental efficiency, improve patient safety, and reduce clinician workload by prioritizing cases based on urgency. The adoption of AI in this context has been explored across several studies, each providing insights into its potential and limitations. This systematic review examines the work efficiency and patient safety implications of AI implementation in ophthalmic emergency departments, whilst exploring feasibility and future directions. A comparative analysis of four recent studies highlights key similarities and differences in their approaches and outcomes.

Safety of AI Triage Implementation in Eye Casualty Departments

Safety is a critical aspect of implementing AI in eye casualties. The study by Chen et al. on the "EE-Explorer" demonstrated that the AI system achieved high accuracy in triaging patients, with an area under the receiver operating characteristic curve (AUC) of 0.988 during external validation, demonstrating reliability to distinguish between urgent and non-urgent cases. Similarly, Brandao-de-Resende et al. reported a sensitivity of 95.2% in external validation, underscoring the system's robustness in correctly identifying high-priority cases.

However, Jindal et al.'s usability study highlighted clinician concerns about potential over-reliance on AI, especially in ambiguous cases [13]. These concerns align with broader literature warning about algorithmic biases and the potential for missed diagnoses in less-represented populations [16]. Despite these limitations, all studies affirmed the general safety of AI systems when integrated with clinician oversight, mitigating risks associated with fully automated decision-making. These findings align with broader research suggesting that AI could enhance triage safety by reducing human error and improving decision-making accuracy [16].

Work Efficiency From AI Triage Implementations

AI triage systems have demonstrated their ability to enhance departmental efficiency. The "EE-Explorer" reduced triage times significantly by automating initial patient assessments. Similarly, the AI-powered platform in Jindal et al.’s study streamlined workflows, with 95% of clinicians reporting reduced time per patient assessment.

Brandao-de-Resende et al.’s ML model also improved efficiency by prioritizing emergency cases, which reduced bottlenecks during peak hours. However, Ahn’s study emphasized that the integration of AI must consider resource availability, as high computational demands could impede real-time implementation in resource-limited settings. Overall, these findings suggest that AI can optimize workflow by reallocating clinician time towards more complex cases, enhancing overall productivity. Additionally, a study of AI integration in the emergency department has been associated with reduced length of hospital stay, hence supporting improved efficiency. This further supports the safe implementation of AI in the emergency eye department [17].

Patient Safety Aspects From AI Eye Casualty Triage Implementation

Patient safety remains a central focus in the implementation of AI in triage. Studies by Chen et al. and Brandao-de-Resende et al. reported high sensitivity in identifying critical cases, ensuring timely referrals and reducing the risk of adverse events. This is in agreement with a study by Cotte et al., where AI applications in triage have demonstrated identical patient triage or have adopted a more conservative approach, compared to traditional methods. This has the benefit of potentially reducing the risk of adverse events [18]. Conversely, concerns about over-triaging in Jindal et al.’s study indicated a need for balancing sensitivity with specificity to avoid unnecessary referrals, which can strain healthcare resources and patient trust.

Ahn’s study further emphasized the importance of integrating AI triage with clinician validation, particularly in ambiguous cases. This aligns with recommendations from similar studies which stress the importance of maintaining clinician oversight to prevent errors associated with over-reliance on algorithms [19]. Collectively, these studies suggest that AI systems may enhance patient safety when used as adjunct tools rather than standalone decision-making.

Feasibility and Future Directions of AI Triage in Eye Casualty

The feasibility of AI implementation depends on its adaptability, clinician acceptance, and resource requirements. Jindal et al.’s study highlighted high clinician acceptance, with 95% of participants willing to incorporate AI into their practice. Additionally, the integration of AI in triage processes has been associated with positive outcomes in various criteria, suggesting its potential to enhance emergency care [20]. A potential avenue for further analysis would potentially be to evaluate current ophthalmic trainees utilizing AI tools to explore the impact of these on their clinical skills and confidence.

However, there are still significant challenges that need to be addressed regarding the implementation of AI in eye casualty and the need for further studies. For instance, Brandao-de-Resende et al. and Ahn noted challenges in ensuring equitable performance across diverse populations, emphasizing the need for broader external validations.

Future directions include refining AI algorithms to handle incomplete or ambiguous data and integrating them with electronic health records for seamless operation. Additionally, expanding validation efforts across geographic and demographic contexts is essential to address potential biases and ensure equitable care delivery.

Limitations

This systematic review aimed to review the current implementation of AI in triaging patients in eye casualty departments. Limitations of this study include the limited number of studies utilized and the time period over which the studies were identified. Both limitations are attributable to the recent advancements in the implementations of AI, highlighting the limited understanding of the potential use of AI in eye casualty departments. A further limitation of our study was restricting articles reviewed to English language-only studies, which could possibly overlook the use of AI in triaging patients in eye casualty departments in other published works.

## Conclusions

A review of the literature provides a limited but promising scope for the implementation of AI in triaging in eye casualty departments. The studies evaluated have helped address our review question and provide further insight into the scope of AI in eye casualty departments. The studies have shown improvement in work efficiency without compromising patient safety. They have demonstrated high sensitivity in identifying critical cases with timely referrals. However, several challenges limit the clinical applicability of AI in eye casualty settings at present, with all studies expressing the importance of further clinical validation and prevention of potential over-reliance on such tools in the future. The importance of further studies will over time allow for more robust external validations of findings and help provide a better understanding of the general applicability of AI tools in triaging in eye casualty settings globally. All studies reviewed provide invaluable insights into this topic. There remains a shared understanding that further studies and evaluation are needed prior to the widespread implementation of AI in triaging patients in eye casualty settings. However, on review of the available studies we would like to propose possible recommendations for assessing the implementation of AI in triaging patients in the eye casualty department. Recommendations include prioritization of AI integration in pilot programs and expanding datasets for external validation to establish the future applicability of AI in triaging patients in eye casualty settings.
